# Endovascular treatment of pulsatile tinnitus associated with venous sinus diverticulum: a scoping review

**DOI:** 10.3389/fsurg.2026.1859104

**Published:** 2026-08-10

**Authors:** Peter Curpen, Shalin Parikh, Kartik Nayak, Chandrashekhar Patel, Jacob Helou, Ramon Navarro

**Affiliations:** 1 Department of Neurosurgery, Princess Alexandra Hospital and Health Service, Brisbane, QLD, Australia; 2 Queensland Brain Institute, University of Queensland, Brisbane, QLD, Australia; 3 Department of Emergency Medicine, Princess Alexandra Hospital and Health Service, Brisbane, QLD, Australia; 4 Faculty of Health Sciences and Medicine, Bond University, Gold Coast, QLD, Australia; 5 Department of Neurology, Townsville Hospital and Health Service, Townsville, QLD, Australia; 6 Department of Neurosurgery, Townsville Hospital and Health Service, Townsville, QLD, Australia

**Keywords:** coiling, endovascular treatment, pulsatile tinnitus, scoping review, sinus diverticulum, stenting

## Abstract

**Background:**

Venous sinus diverticula are a recognised and potentially treatable cause of pulsatile tinnitus. While surgical resurfacing has traditionally been employed, a range of endovascular approaches—including coil embolization, venous sinus stenting, and intrasaccular devices—have emerged as less invasive alternatives. The scope and quality of evidence supporting these interventions, however, remain poorly defined.

**Materials and methods:**

PubMed, Embase, Scopus, and Web of Science were systematically searched for studies reporting endovascular treatment of pulsatile tinnitus caused by venous sinus diverticula. Eligible observational studies, case series, and case reports were selected using a Population–Concept–Contextframework. Data extraction and screening were independently performed by multiple reviewers, and findings were synthesised descriptively with quality appraisal to contextualise the evidence.

**Results:**

A total of 23 studies comprising 78 patients were included, predominantly single-centre case reports (*n* = 18) and case series (*n* = 3), with two retrospective cohort studies. Patients were mainly female (84%), with a mean age of 45 years and a mean symptom duration of 4.6 years. Pulsatile tinnitus was most frequently right-sided (53.8%), followed by left-sided (26.9%) and bilateral symptoms (19.2%). Endovascular strategies included stent-assisted coil embolization (60.0%), venous sinus stenting alone (22.5%), coil embolization alone (12.5%), and, less commonly, intrasaccular devices or balloon-assisted techniques (each 2.5%). Complete resolution of symptoms was reported in 84.4% of patients, partial improvement in 10.4%, and no improvement in 5.2%. Follow-up was reported in 19 studies, ranging from 4 weeks to 2 years. Procedural complications were uncommon and generally transient, with no reported mortality or permanent neurological morbidity.

**Conclusion:**

Endovascular treatment shows high symptomatic response rates in selected patients with venous sinus diverticulum–related pulsatile tinnitus, but evidence is limited by small retrospective studies and heterogeneous reporting of outcomes. Prospective studies with standardised diagnostics and validated tinnitus measures are required.

## 1 Introduction

Pulsatile tinnitus (PT) is a distinct subtype of tinnitus characterised by a rhythmic sound synchronised with the cardiac cycle. Although less common than non-pulsatile tinnitus, PT is clinically significant because of its persistent and intrusive nature and its frequent association with identifiable—and potentially treatable—structural pathology. Patients with PT often experience substantial symptom burden, underscoring the importance of timely diagnosis and targeted intervention (1).

The aetiology of PT is heterogeneous, encompassing both vascular and non-vascular causes. Venous abnormalities are increasingly recognised contributors, accounting for an estimated 4%–20% of cases, with venous sinus diverticula and associated venous sinus stenosis among the most commonly implicated lesions (2, 3). Venous sinus diverticula are focal outpouchings or defects of the dural venous sinus wall, most frequently involving the sigmoid sinus (4). Although their precise origin remains debated, emerging haemodynamic and imaging evidence suggests that these lesions may be acquired, arising from turbulent flow, elevated venous pressure, and increased wall shear stress, often in the setting of upstream venous sinus stenosis (3). Such haemodynamic disturbances are thought to generate aberrant venous sound and facilitate its transmission to the inner ear, resulting in pulse-synchronous symptoms (5).

Management strategies for PT associated with venous sinus diverticulum include conservative, surgical, and endovascular approaches. Conservative measures may be appropriate in selected patients, particularly when symptoms are mild or intracranial hypertension is suspected (6). Surgical treatment, most commonly transmastoid sigmoid sinus wall reconstruction, has historically demonstrated high rates of symptom resolution in appropriately selected cases (6). More recently, minimally invasive endovascular techniques, including coil embolization, venous sinus stenting, stent-assisted coiling, and intrasaccular embolization devices, have emerged as alternative strategies aimed at modifying pathological venous haemodynamics or excluding the diverticulum (4, 6).

Despite increasing clinical adoption, the evidence supporting endovascular management remains fragmented. The existing literature is dominated by small, single-centre case reports and case series, with heterogeneous methodologies, variable follow-up durations, and limited comparative data between treatment strategies (7, 8). Consequently, consensus regarding optimal patient selection and procedural approach remains lacking.

Accordingly, this scoping review aims to systematically synthesise the available literature on endovascular management of PT associated with venous sinus diverticulum. By mapping reported anatomical and imaging characteristics, endovascular techniques, clinical outcomes, and procedural complications, this review seeks to clarify the current evidence base and identify priority areas for future research to support the development of more standardised diagnostic and management frameworks.

## 2 Materials and methods

### 2.1 Study design

This scoping review was undertaken in line with the Preferred Reporting Items for Systematic Reviews and Meta-Analyses extension for Scoping Reviews (PRISMA-ScR) (9). The review process was guided by the scoping review methodology originally proposed by Arksey and O'Malley and subsequently refined by later methodological developments (10). This approach involved a structured, iterative process encompassing five key stages: (I) formulation of the research question; (II) identification of relevant evidence; (III) study selection; (IV) data charting and extraction; and (V) the synthesis and presentation of findings.

### 2.2 Protocol registration

The final protocol was registered with the International Platform of Registered Systematic Review and Meta-Analysis Protocols (INPLASY) on 12/1/2026 (INPLASY202610037).

### 2.3 Search strategy and study selection

A systematic and comprehensive search of the literature was performed across four electronic databases: PubMed, Embase, Scopus, and Web of Science. The search strategy was designed to identify studies relating to sigmoid sinus diverticulum and endovascular treatment approaches, incorporating relevant keywords and indexed terms specific to each database.

The inclusion criteria were guided by the Population–Concept–Context (PCC) framework recommended by the Joanna Briggs Institute for scoping reviews (11, 12). Specifically, studies were included if they met the following criteria: (I) Population: patients diagnosed with PT attributable to sinus diverticulum; (II) Concept: endovascular treatment of PT, including transvenous or endovascular embolization and stent-based techniques treating the diverticulum directly or stenosis that has caused the diverticulum; (III) Context: any clinical or interventional setting in which such treatments were delivered; (IV) peer-reviewed publications reporting original empirical data; (V) studies published in English (or with an English translation); and (VI) availability in full-text form. Eligible study designs included randomized controlled trials, observational studies, case series, and case reports.

Numerous studies were excluded during the full-text review if they met any of the following criteria: (I) studies focusing exclusively on surgical (non-endovascular) management of PT associated with sinus diverticula; (II) studies examining alternative venous pathologies or isolated idiopathic intracranial hypertension as the primary cause of PT; (III) studies lacking empirical data; (IV) letters to the editor, commentaries, editorials, or opinion-based articles; (V) grey literature, including clinical guidelines and government reports; (VI) non-English language studies; (VII) or studies unavailable in full-text form. No restrictions were applied to the date of publication to maximise capture of the available literature. In addition to database searches, manual search strategies were undertaken, including hand-searching reference lists of included studies and citation tracking to identify additional relevant publications.

Records retrieved from the database searches were imported into Covidence, a systematic review management platform, where duplicate entries were automatically identified and removed. Screening was conducted in two stages: an initial assessment of titles, keywords, and abstracts to determine relevance, followed by full-text review of potentially eligible studies against predefined inclusion and exclusion criteria. Both stages of screening were performed independently by two reviewers (KN and CP). Discrepancies were resolved through discussion, with arbitration by a third reviewer (PC) when necessary until consensus was reached.

### 2.4 Data extraction

Data extraction was independently performed by three reviewers (KN, CP, and PC) using a predefined data extraction template. Extracted study-level variables included author(s), year of publication, country, study design, and sample size. Patient-level characteristics, where reported, included age, sex, and relevant clinical comorbidities such as obesity or idiopathic intracranial hypertension. Radiological variables were then recorded, including sinus diverticulum morphology (e.g., saccular or post-stenotic dilatation), diverticulum location and dimensions, presence of sigmoid plate dehiscence, and associated transverse sinus stenosis or venous pressure gradients. Procedural details extracted included the type of endovascular intervention performed, such as venous sinus stenting, coiling, or stent-assisted coiling. Outcome-related variables, where available, included duration of PT prior to intervention, length of hospital stay, follow-up duration, symptomatic response, and reported procedural or post-procedural complications. Discrepancies identified during the data extraction process were resolved through discussion with the senior author (RN) until consensus was achieved.

### 2.5 Data analysis

Data from included studies on endovascular treatment of PT associated with venous sinus diverticulum were systematically collated and synthesised using descriptive and thematic approaches. Quantitative data were summarised using frequencies and proportions to characterise patient demographics, diverticulum and venous sinus characteristics, endovascular techniques employed (e.g., coiling, stenting, or combined approaches), procedural success, complication rates, and tinnitus outcomes. Qualitative data were analysed thematically and grouped into clinically relevant categories, including treatment selection rationale, imaging features, symptom resolution, recurrence, and follow-up duration. Key results were presented using structured tables and graphical summaries where appropriate.

### 2.6 Literature quality assessment

Although a formal risk-of-bias assessment is not mandatory for scoping reviews, a structured evaluation of methodological quality was undertaken to improve transparency and facilitate interpretation of the evidence. Two reviewers (SP and CP) independently appraised all included studies using design-appropriate assessment tools. Retrospective cohort studies were evaluated using the Newcastle–Ottawa Quality Assessment Scale (13). Case series and case reports, on the other hand, were assessed using an adapted version of the Newcastle–Ottawa framework that incorporates additional domains from the Pierson and Bradford Hill criteria, as described by Murad and colleagues, to address methodological features specific to descriptive study designs (14).

Each appraisal tool comprised eight assessment domains; items considered not applicable for a particular study were excluded from the total score denominator. Study quality was categorised as high (>80% of applicable criteria met), moderate (60%–80%), or low (<60%). Disagreements between reviewers were resolved by consensus, with adjudication by a third reviewer (PC) where required. Quality ratings were documented alongside extracted study data, and all studies were retained for evidence synthesis regardless of appraisal outcome, consistent with the objectives of a scoping review.

## 3 Results

### 3.1 Study selection

**Figure 1:**
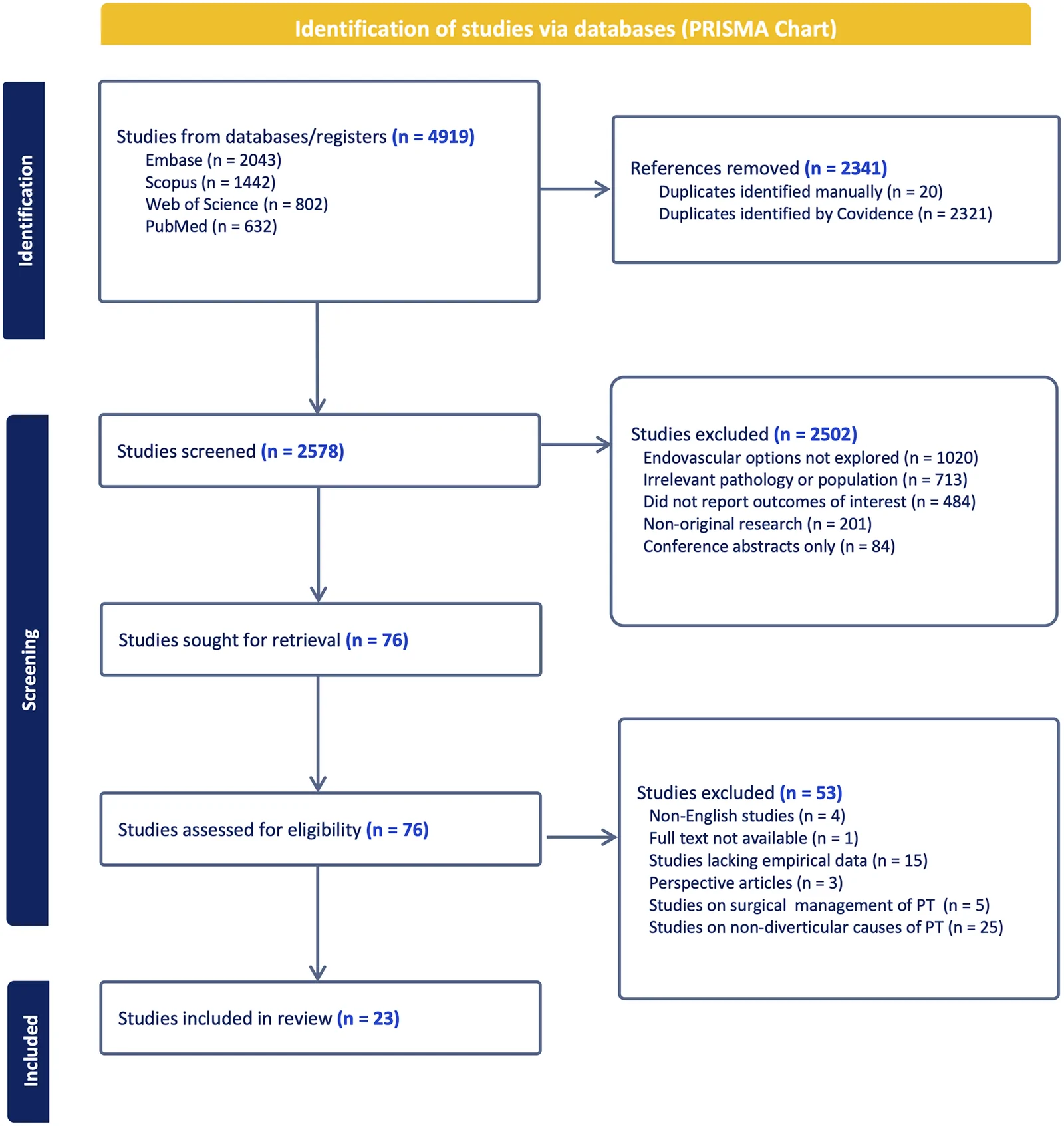
PRISMA flow diagram with screening numbers.

The literature search, conducted on 21 December 2025, identified 4,919 records across PubMed, Embase, Scopus, and Web of Science. After removal of duplicate records, 2,578 unique studies remained. Title and abstract screening resulted in 76 studies proceeding to full-text review. Of these, 23 studies met the inclusion criteria and were included in the final analysis (Figure 1).

### 3.2 Characteristics of included studies

The characteristics of the 23 included studies are summarised in Table 1. In total, 23 peer-reviewed studies comprising 78 patients were included, consisting of 2 retrospective cohort studies, 18 case reports, and 3 case series. All studies were single-centre in design and originated from multiple countries, most commonly the United States (*n* = 10), followed by Germany (*n* = 2), France (*n* = 2), China (*n* = 2), Brazil (*n* = 2), Mexico (*n* = 2), Italy (*n* = 1), South Korea (*n* = 1), and Canada (*n* = 1). Sample sizes ranged from 1 to 26 patients. Based on the applied methodological appraisal criteria, 16 studies were rated as high quality and 7 as moderate quality (Tables 2, 3).

**Table 1:** Characteristics of included studies.study characteristics of included articles reporting pulsatile tinnitus associated with sinus diverticula.

Author (Year)	Study title	Country	Study Design	Primary Data Source	Recruitment period	Sample Size	Quality assessment
Abdalkader et al. (3)	Venous sinus diverticulum in patients with pulsatile tinnitus: An acquired lesion.	United States of America	Case Report	Electronic Medical Records	N/A	1	Moderate
Abdalkader et al. (15)	Endovascular treatment of sigmoid Sinus diverticulum in patients with pulsatile tinnitus.	United States of America	Single-centre, Retrospective observational study	Electronic medical Records	Sep 2020– Jan 2024	26	Moderate
Abozenah et al. (16)	Successful treatment of pulsatile tinnitus caused by jugular bulb diverticula using stent-assisted volumetric coil embolization in a double-jailing technique	Germany	Case Report	Electronic medical records	N/A	1	Moderate
Amans et al. (17)	Resolution of pulsatile tinnitus after coil embolization of sigmoid sinus diverticulum	United States of America	Case Report	Electronic medical records	N/A	1	High
Cuellar et al. (18)	Endovascular Treatment of Pulsatile Tinnitus by Sigmoid Sinus Aneurysm: Technical Note and Review of the Literature.	United States of America	Technical Note and Literature Review	Electronic medical records	N/A	1	Moderate
Drescher et al. (19)	Pulsatile tinnitus due to an aneurysmatic diverticulum of the jugular bulb treated with the Woven EndoBridge device	Germany	Case Report	Electronic medical records	N/A	1	High
Gard et al. (20)	Successful endovascular treatment of pulsatile tinnitus caused by a sigmoid sinus aneurysm. A case report and review of the literature.	United States of America	Case Report	Electronic medical records	N/A	1	High
Houdart et al. (21)	Aneurysm of a dural sigmoid sinus: a novel vascular cause of pulsatile tinnitus.	France	Case Report	Electronic medical records	N/A	1	High
Keshary et al. (22)	Stent-assisted coiling of dural sinus diverticula: a case series	United States of America	Case Series	Electronic medical records	N/A	8	High
Lenck et al. (23)	Pulsatile tinnitus caused by an aneurysm of the transverse-sigmoid sinus: a new case report and review of literature.	France	Case Report and Literature Review	Electronic medical records	N/A	1	High
Li et al. (24)	Stent-Assisted Coil Embolization of a Transverse-Sigmoid Sinus Diverticulum Presenting with Pulsatile Tinnitus.	China	Case Report	Electronic medical records	N/A	1	High
Mehanna et al. (25)	Endovascular treatment of sigmoid sinus aneurysm presenting as devastating pulsatile tinnitus. A case report and review of literature.	United States of America	Case Report and Literature Review	Electronic medical records	N/A	1	High
Paramasivam et al. (26)	Endovascular Management of Sigmoid Sinus Diverticulum	United States of America	Case Report	Electronic medical records	N/A	1	High
Park et al. (27)	Awake embolization of sigmoid sinus diverticulum causing pulsatile tinnitus: simultaneous confirmative diagnosis and treatment.	South Korea	Case Report	Electronic medical records	N/A	1	High
Qiu et al. (28)	Bone remodeling in sigmoid sinus diverticulum after stenting for transverse sinus stenosis in pulsatile tinnitus: A case report.	China	Case Report	Electronic medical records	N/A	1	Moderate
Sanchez et al. (29)	A new therapeutic procedure for treatment of objective venous pulsatile tinnitus.	Brazil	Case Report	Electronic medical records	N/A	1	High
Santos-Franco et al. (30)	Hybrid Carotid Stent for the Management of a Venous Aneurysm of the Sigmoid Sinus Treated by Sole Stenting	Mexico	Case Report	Electronic medical records	N/A	1	High
Shastri et al. (31)	Venous Diverticula Causing Pulsatile Tinnitus Treated with Coil Embolization and Stent Placement with Resolution of Symptoms: Report of Two Cases and Review of the Literature.	United States of America	Case Series	Electronic medical records	N/A	2c	High
Signorelli et al. (32)	Endovascular treatment of two concomitant causes of pulsatile tinnitus: sigmoid sinus stenosis and ipsilateral jugular bulb diverticulum. Case report and literature review.	Italy	Case report and literature review	Electronic medical records	N/A	1	High
Trivelato et al. (33)	Endovascular treatment of pulsatile tinnitus associated with transverse sigmoid sinus aneurysms and jugular bulb anomalies.	Brazil	Case Report and literature review	Electronic medical records	N/A	1	High
Walker et al. (4)	Clinical presentation and patient outcomes following endovascular intervention for venous sinus diverticula: A single center experience.	United States of America	Single-centre, Retrospective observational study	Electronic medical records	2019−2023	23	High
Zenteno et al. (34)	Endovascular treatment of a transverse-sigmoid sinus aneurysm presenting as pulsatile tinnitus. Case report.	Mexico	Case Report	Electronic medical records	N/A	1	High
Zur et al. (35)	Stent-assisted Woven EndoBridge embolization for the treatment of pulsatile tinnitus caused by an ipsilateral high-riding jugular bulb.	Canada	Case Series	Electronic medical records	N/A	1	Moderate

The table summarises author, year of publication, study design, data source, recruitment period, sample size, country of origin, and methodological quality. Included studies were predominantly case reports and case series, with two single-centre retrospective observational studies, and all derived data from local medical records across diverse international settings.

**Table 2:** Risk of bias assessment of included retrospective studies.

Newcastle Ottawa Assessment scale—Retrospective observational studies											
Author (Year)	Selection				Comparability		Outcome			Total	Quality assessment
	Representativeness of exposed cohort	Selection of non-exposed cohort	Ascertainment of exposure	Outcome not initially present	Study controls main confounder	Study controls for additional confounder	Assessment of outcome	Follow up long enough for outcomes to occur?	Adequate follow up?		
Abdalkader et al. (15)	★	0	★	★	★	★	★	★	0	7/9	Moderate
Walker et al. (4)	★	0	★	★	★	★	★	★	★	8/9	High

This table summarises the methodological quality and risk of bias of included retrospective observational studies evaluating endovascular management of pulsatile tinnitus secondary to venous sinus diverticula, assessed using the Newcastle–Ottawa Quality Assessment Scale. Studies were independently appraised by two reviewers across the domains of Selection, Comparability, and Outcome. A star was awarded when predefined criteria were met, with a maximum of one star per item in the Selection and Outcome domains and up to two stars in the Comparability domain. Domain scores were aggregated to generate an overall study quality rating of low, moderate, or high.

**Table 3:** Risk of bias assessment of included case reports and case series.

Newcastle Ottawa Assessment scale—Case reports and Case series									
Author (Year)	Clear case definition	Representativeness	Exposure ascertainment	Outcome ascertainment	Temporality	Plausibility	Consistency	Adequate follow up?	Quality assessment
Abdalkader et al. (3)	High	Moderate	High	High	High	Moderate	N/A	Low	Moderate
Abozenah et al. (16)	High	Moderate	High	High	High	High	N/A	Low	Moderate
Amans et al. (17)	High	Moderate	High	High	High	Moderate	N/A	High	High
Cuellar et al. (18)	High	Moderate	High	Moderate	High	Moderate	N/A	Low	Moderate
Drescher et al. (19)	High	Moderate	High	High	High	Moderate	N/A	High	High
Gard et al. (20)	High	Moderate	High	High	High	Moderate	N/A	High	High
Houdart et al. (21)	High	High	High	High	High	High	N/A	High	High
Keshary et al. (22)	High	High	High	High	High	High	High	High	High
Lenck et al. (23)	High	High	High	High	High	High	N/A	High	High
Li et al. (24)	High	High	High	High	High	High	N/A	Moderate	High
Mehanna et al. (25)	High	High	High	High	High	High	N/A	High	High
Paramasivam et al. (26)	High	High	High	High	High	High	N/A	High	High
Park et al. (27)	High	High	High	High	High	High	N/A	High	High
Qiu et al. (28)	High	High	High	Moderate	High	Moderate	N/A	High	Moderate
Sanchez et al. (29)	High	High	High	High	High	High	N/A	High	High
Santos-Franco et al. (30)	High	High	High	High	High	High	N/A	High	High
Shastri et al. (31)	High	High	High	High	High	High	High	High	High
Signorelli et al. (32)	High	Moderate	High	High	High	High	N/A	High	High
Trivelato et al. (33)	High	High	High	High	High	High	N/A	High	High
Zenteno et al. (34)	High	High	High	High	High	High	N/A	High	High
Zur et al. (35)	High	Moderate	High	High	High	Moderate	High	High	Moderate

This table summarises the methodological quality and risk of bias of the included case reports and case series, using an adapted Newcastle–Ottawa framework that incorporates additional domains from the Pierson and Bradford Hill criteria. Studies were independently assessed by two reviewers across eight domains: case definition, representativeness, temporality, plausibility, consistency, follow-up, and exposure and outcome ascertainment. Based on these domains, each study was assigned an overall methodological quality rating of low, moderate, or high.

### 3.3 Patient characteristics

The patient characteristics of the included studies are summarised in Table 4. PT attributable to venous sinus diverticula predominantly affected females (84%; *n* = 63). Patients were typically middle-aged, with a mean age of 45 years. Symptoms were right-sided in 53.8% of cases (*n* = 42), left-sided in 26.9% (*n* = 21), and bilateral in 19.2% (*n* = 15). Duration of PT was reported in 19 studies, with a mean symptom duration of 4.6 years prior to intervention. Tinnitus severity was documented in 14 studies and was most commonly described as moderate to severe where reported. Commonly associated symptoms and comorbid conditions included headache (*n* = 23), visual disturbance (*n* = 13), obesity (*n* = 14), and idiopathic intracranial hypertension (*n* = 20), which were inconsistently reported across studies.

**Table 4:** Patient demographic and clinical characteristics.

Patient characteristics of included articles reporting pulsatile tinnitus associated with sinus diverticula								
Author (Year)	Age (years)	Sex	Laterality of pulsatile tinnitus	Duration of symptoms	Tinnitus severity	Associated symptoms	Prior treatment	Relevant comorbidities
Abdalkader et al. (3)	N/A	Female	Right	17 years	Severe	None	Yes (topiramate, acetazolamide, gabapentin, sumatriptan, amitriptyline, acupuncture)	Yes (Migraine, Meniere's disease, idiopathic intracranial hypertension, gastric bypass)
Abdalkader et al. (15)	Median age 40	Female *n* = 25	Left *n* = 5; Right *n* = 16; Bilateral *n* = 5 cases	Median duration 31 months	Moderate severity in 3 cases; High severity in 23 cases	Headache *n* = 18; Blurred vision *n* = 9; Other visual disturbances *n* = 7; Hearing loss *n* = 6	None	Idiopathic intracranial hypertension *n* = 17 Obesity *n* = 12
		Male *n* = 1						
Abozenah et al. (16)	40–49	N/A	Right	N/A	Severe	None	Unsuccessful initial treatment for presumed Eustachian tube dysfunction	None
Amans et al. (17)	59	Female	Right	18 months	Severe	None	None	None
Cuellar et al. (18)	56	Male	Right	3 months	Severe	Loss of consciousness 3 months prior; occasional right sided otorrhagia; prior right mastoid infection	None	Diabetes, Hypertension, Obstructive Sleep Apnoea
Drescher et al. (19)	68	Female	Left	5 months	N/A	Headaches	None	None
Gard et al. (20)	48	Female	Left	Several months (unclear duration)	Severe	Headaches	None	None
Houdart et al. (21)	33	Female	Left	6 months	Severe	None	None	None
Keshary et al. (22)	N/A	Female n = 8 Male *n* = 1	Right *n* = 5; Left *n* = 2; Bilateral *n* = 1	N/A	N/A	Headaches *n* = 2	None	Idiopathic intracranial hypertension *n* = 1
Lenck et al. (23)	28	Female	Right	N/A	Severe	Sleep disturbances and depression	None	None
Li et al. (24)	39	Female	Right	4 years	Severe	None	None	None
Mehanna et al. (25)	46	Female	Right	6 years	Severe, worsening progression	Aural pain	None	None
Paramasivam et al. (26)	N/A	Female	Right	4 months	N/A	None	None	Obesity
Park et al. (27)	31	Female	Right	3 years	N/A	None	None	None
Qiu et al. (28)	45	Male	Left	15 years	N/A	Dizziness, Headaches	Surgeries for nasal septum reconstruction and intraocular lens implantation 9 years prior	Idiopathic intracranial hypertension
Sanchez et al. (29)	54	Male	Left	3 years	Severe	None	None	None
Santos-Franco et al. (30)	59	Female	Bilateral	20 years	Severe	Sleep disturbances, suicidal ideation	None	None
Shastri et al. (31)	Case 1: 27	Case 1: Female Case 2: Female	Case 1: Right	Case 1: 3 months Case 2: 3 months	N/A	N/A	Case 1: N/A	Case 1: N/A
	Case 2: 29		Case 2: Right				Case 2: suboccipital decompression 2 years prior	Case 2: History of Chiari I malformation
Signorelli et al. (32)	59	Female	Right	4 years	Severe	Aural pain	None	Mild obesity
Trivelato et al. (33)	45	Female	Left	2 years	Incapacitating/Severe	None	None	None
Walker et al. (4)	Mean age 54.65	Female *n* = 16 Male *n* = 7	Right *n* = 8; Left *n* = 7; Midline *n* = 8	N/A	N/A	N/A	N/A	N/A
Zenteno et al. (34)	38	Female	Left	2 months	N/A	N/A	An index procedure involving a burr hole to allow for the placement of a wallstent over the aneurysm	None
Zur et al. (35)	N/A	N/A	Right	10 years	N/A	N/A	N/A	None

This table demonstrates the demographic characteristics of patients across included studies, including age, sex, laterality of pulsatile tinnitus symptoms, symptom duration, and reported tinnitus severity. Additional domains include prior treatments, associated symptoms, and comorbid conditions, providing context for the clinical burden of pulsatile tinnitus. Across studies, tinnitus was commonly described as severe or incapacitating and was frequently accompanied by headache, sleep disturbance, and mood symptoms. Reported comorbidities included idiopathic intracranial hypertension, hypertension, obesity, and diabetes.

### 3.4 Anatomic and imaging characteristics of venous sinus diverticula

Table 5 summarises the anatomical and imaging characteristics of venous sinus diverticula across the included studies. A total of 79 diverticula were reported, most commonly involving the transverse–sigmoid sinus junction (32.9%) and sigmoid sinus (29.1%), followed by the lateral sinus (20.3%), jugular bulb (16.5%), and transverse sinus (1.3%). Laterality was reported in all studies, with a strong right-sided predominance (64.56%, *n* = 51), compared with left-sided diverticula (30.38%, *n* = 24) with 4 cases of bilateral involvement (5.06%). Bilateral lesions were uncommon and should not be interpreted as equivalent to cases presenting with bilateral pulsatile tinnitus, as symptom laterality was reported separately and was not consistently related to lesion laterality. Sinus dominance was described in 13 studies (*n* = 35), with right-sided dominance most frequently observed (*n* = 20), followed by left-sided dominance (*n* = 10) and co-dominance (*n* = 5).

**Table 5:** Imaging and structural characteristics of venous sinus diverticula.

Radiological characteristics of included articles reporting pulsatile tinnitus associated with sinus diverticula									
Author (Year)	Diverticula location	Diverticula laterality	Diverticula size	Diverticula Shape	Associated Sinus stenosis?	Other Venous anomalies	Dominant sinus	Associated bone wall abnormalities?	Physiologic testing
Abdalkader et al. (3)	Transverse sinus	Right	7 mm × 6 mm × 5 mm	Saccular	Yes	None	N/A	Sigmoid sinus plate dehiscence	N/A
Abdalkader et al. (15)	Transverse-Sigmoid sinus junction *n* = 20	Right *n* = 16	N/A	Post-stenotic dilatation *n* = 6 Saccular *n* = 20	Yes, in 6 cases	None	N/A	Dehiscence in 22 cases	N/A
	Sigmoid Sinus *n* = 6	Left *n* = 8							
		Bilateral *n* = 2							
Abozenah et al. (16)	Jugular bulb *n* = 2	Right *n* = 2	N/A	N/A	No	Enlarged right nuchal vein outlet	N/A	None	Balloon test with resolution of tinnitus on inflation
Amans et al. (17)	Sigmoid Sinus	Right	6.8 mm×8.0 mm×4.2 mm	Lobular	Yes	None	Right	marginated scalloping of the inner table of the sigmoid plate	N/A
Cuellar et al. (18)	Sigmoid Sinus	Right	N/A	N/A	No	None	N/A	None	N/A
Drescher et al. (19)	Jugular Bulb	Left	6 mm×7 mm	N/A	No	None	N/A	None	N/A
Gard et al. (20)	Sigmoid Sinus	Left	6 mm×6mm	N/A	No	Enlargement of Left Jugular Bulb	Left	Dehiscence of posterosuperior aspect of the mastoid region	N/A
Houdart et al. (21)	Sigmoid Sinus	Left	N/A	N/A	No	None	Left	Smooth cavity in the left temporal bone	N/A
Keshary et al. (22)	Sigmoid Sinus (*n* = 8)	Right *n* = 5	Mean dimensions of all diverticula: 6.62 mm×6.16 mm	Saccular *n* = 2 Multilobulated *n* = 2	Yes, *n* = 6	N/A	N/A	Case 4: Dehiscence of sigmoid sinus plate	Fusiform *n* = 2
		Left *n* = 2		Bilobed *n* = 2	No, *n* = 2			Case 6: Erosion through the mastoid cortex	N/A
				Flame *n* = 1					
Lenck et al. (23)	Transverse Sigmoid sinus junction	Right	N/A	N/A	No	None	Right	Bony defect in the posterior wall of the right petrous bone	N/A
Li et al. (24)	Transverse Sigmoid Sinus junction	Right	N/A	N/A	No	None	Right	Erosion of mastoid bone	N/A
Mehanna et al. (25)	Sigmoid Sinus	Right	7.4 mm×4mm	N/A	No	High riding right jugular bulb	Right	N/A	N/A
Paramasivam et al. (26)	Sigmoid Sinus	Right	7 mm×5 mm	N/A	Yes	Hypoplasia of left transverse-sigmoid sinus	Right	Dehiscence of sigmoid sinus plate	N/A
Park et al. (27)	Sigmoid Sinus	Right	7 mm×6 mm	N/A	No	None	Right	Defect of temporal bone	N/A
Qiu et al. (28)	Sigmoid Sinus	Left	N/A	N/A	Yes	Contralateral Transverse sinus dysplasia	Left	Dehiscence of sigmoid sinus plate	N/A
Sanchez et al. (29)	Transverse Sigmoid sinus junction	Left	N/A	N/A	No	None	Left	N/A	N/A
Santos-Franco et al. (30)	Sigmoid Sinus	Right	N/A	N/A	Yes	None	N/A	Dehiscence of sigmoid sinus plate	N/A
Shastri et al. (31)	Transverse Sigmoid sinus junction *n* = 2	Right *n* = 2	Case 1: 10 × 8 × 10 mm	N/A	N/A	N/A	N/A	Case 1: Dehiscence of temporal bone	N/A
			Case 2: 16 × 8 × 10 mm					Case 2: Dehiscence of sigmoid sinus plate	
Signorelli et al. (32)	Jugular bulb	Right	4 mm diameter	N/A	Yes	N/A	N/A	Erosion of bony septum that comprises the floor of the tympanic sinus	Balloon test with resolution of tinnitus on inflation
Trivelato et al. (33)	Jugular bulb	Left	N/A	N/A	Yes	Transverse sinus hypoplasia, high riding jugular bulb	Left	Erosion of bony septum + Enlargement of jugular foramen	N/A
Walker et al. (4)	Lateral sinus *n* = 16	Left *n* = 7	N/A	N/A	Yes, in 7 cases	N/A	Right *n* = 14	Bony dehiscence *n* = 11	N/A
	Jugular bulb *n* = 7	Right *n* = 15					Left *n* = 4		
		Bilateral *n* = 1					Co-dominant *n* = 5		
Zenteno et al. (34)	Transverse Sigmoid Sinus junction	Left	8 mm × 6mm	N/A	N/A	N/A	Left	Left petrous bone erosion at Transverse sinus	N/A
Zur et al. (35)	Jugular bulb	Right	N/A	N/A	N/A	High riding jugular bulb	N/A	N/A	Balloon test with resolution of tinnitus on inflation

This table summarises the imaging and structural features of venous sinus diverticula across the included studies. Reported variables include diverticular location, laterality, size, and morphology, as well as associated venous anomalies, venous sinus stenosis, and adjacent bony wall abnormalities. Dominant venous drainage patterns and, where reported, physiological assessment such as balloon occlusion testing are also presented, highlighting the relationship between structural abnormalities and clinical symptoms. Although diverticular morphology varied across studies, lesions were most commonly located at the transverse–sigmoid sinus junction or jugular bulb and were frequently associated with venous sinus stenosis or sigmoid sinus plate dehiscence.

Diverticular morphology was reported in seven studies (*n* = 40), with saccular configuration being most common (*n* = 23), followed by post-stenotic dilatation (*n* = 6); other morphologies were rare and included aneurysmatic, multilobulated, bilobed, fusiform, lobular, flame-shaped, and irregular forms. Diverticular size was reported in 11 studies with a mean measurement of 7.98 mm × 6.18 mm × 6.64 mm. Associated venous sinus stenosis was reported in 10 studies (*n* = 26). Additional venous abnormalities were infrequently reported and included high-riding jugular bulbs (*n* = 4), transverse sinus hypoplasia (*n* = 2), contralateral transverse sinus dysplasia (*n* = 1), and an enlarged nuchal vein outlet (*n* = 1). Bony wall abnormalities were reported in 17 studies, with dehiscence being most common (*n* = 38). Balloon occlusion testing was performed in three studies, with transient symptom resolution reported in all cases.

### 3.5 Endovascular treatment strategies and clinical outcomes

Table 6 summarises the endovascular treatment strategies and clinical outcomes reported for PT associated with venous sinus diverticulum. Coil embolization was required in 12.5% (*n* = 10) of cases, stent assisted coil embolization was required in 60% (*n* = 48) of patients, while other endovascular interventions included stenting in 22.5% (*n* = 18), intrasaccular devices in 2.5% (*n* = 2) and balloon assisted coiling in 2.5% (*n* = 2) of cases. In one case, a two-stage endovascular technique was employed with initial stenting followed by coil embolization as pulsatile tinnitus re-appeared 3 months after the initial procedure.

**Table 6:** Endovascular treatment strategies and clinical outcomes.

Endovascular treatment strategies of included articles reporting pulsatile tinnitus associated with sinus diverticula								
Author (Year)	Endovascular technique	Technical parameters	Peri-procedural management	Length of hospital stay	Treatment efficacy and symptom resolution	Follow up interval	Follow up outcome	Complications
Abdalkader et al. (3)	Coiling, stenting and stent-assisted coiling	N/A	N/A	N/A	Immediate symptom resolution post stent-assisted coiling	N/A	N/A	None
Abdalkader et al. (15)	Stent-assisted coiling *n* = 9	N/A	N/A	N/A	Full resolution at 24 h	3 months and 1 year follow up	Full resolution at 3 months *n* = 23	Access site haematoma *n* = 1
	Coiling *n* = 3				*n* = 23		Partial resolution at 3 months *n* = 3	Coil stretching *n* = 2
	Stenting *n* = 14				Partial resolution at 24 h *n* = 3			
Abozenah et al. (16)	Stent-assisted coiling	Femoral access; systemic intravenous heparinisation.	Prasugrel and Aspirin 5 days pre-procedure. (DAPT) for 12 weeks post procedure, aspirin monotherapy thereafter.	N/A	Immediate symptom resolution	Follow up interval not documented	Complete PT resolution	None
Amans et al. (17)	Coiling only	Femoral access; systemic intravenous heparinisation.	Aspirin daily for 2 weeks post-procedure.	N/A	Immediate symptom resolution	6 months	Complete PT resolution	None
Cuellar et al. (18)	Stent-assisted coiling	Femoral and IJV access; No comment on heparinisation.	Clopidogrel for 5 days. Post-procedure DAPT for 6 months. Thereafter, Aspirin indefinitely.	N/A	Immediate symptom resolution	N/A	N/A	None
Drescher et al. (19)	Woven Endobridge (intrasaccular device)	Femoral access; systemic intravenous heparinisation.	None	2 days	Complete symptom resolution at 5 days	2 months	Complete PT resolution. Headaches remained.	None
Gard et al. (20)	Coiling only	Femoral access and systemic intravenous heparinisation.	Aspirin for 6 weeks post-procedurally.	1 day	Immediate symptom resolution	1 year	Complete PT resolution	None
Houdart et al. (21)	Coiling only	IJV access; No comment on heparinisation.	Aspirin 5 days prior and 1-month post-procedure.	N/A	Immediate symptom resolution	8 months	Complete PT resolution	None
Keshary et al. (22)	Stent-assisted coiling (*n* = 8)	Femoral access in all cases; systemic intravenous heparinisation in all cases.	DAPT for 7 days prior to 6 months post procedure. Thereafter, Aspirin lifelong.	N/A	Symptom resolution *n* = 7	6-week clinical review and 6 month venogram	Full resolution *n* = 7	None
					Partial resolution *n* = 1		Partial resolution *n* = 1	
Lenck et al. (23)	Coiling only	IJV access; no comment on heparinisation	Aspirin for 3 months post-procedure.	2 days	Immediate symptom resolution	7 years	Complete PT resolution	None
Li et al. (24)	Stent-assisted coiling	Femoral access; systemic intravenous heparinisation.	DAPT for 3 days pre-procedure to 1 month post. Thereafter aspirin alone for 6 months.	N/A	Immediate symptom resolution	4 months	Complete PT resolution	None
Mehanna et al. (25)	Balloon assisted coiling	Femoral access; systemic intravenous heparinisation	Aspirin daily for 2 weeks post-procedure.	N/A	80% improvement in PT	6 months and 1 year	6 months: stable improvement in PT	None
							1 year: 90% improvement in PT	
Paramasivam et al. (26)	Coiling only	N/A	DAPT pre-procedurally (unclear duration).	N/A	Immediate symptom resolution	8 months	Complete PT resolution	None
Park et al. (27)	Coiling only	Femoral access; no comment on heparinisation.	None	3 days	Immediate symptom resolution	22 months	Complete PT resolution	None
Qiu et al. (28)	Stenting	N/A	Anticoagulation (unclear type and duration) post-procedurally.	7 days	Immediate symptom resolution	6 months, 1 year and 2 years	N/A	In-stent thrombosis post cessation of anticoagulation
Sanchez et al. (29)	Stent-assisted coiling	IJV access; no comment on heparinisation.	N/A	N/A	Immediate symptom resolution	12 months	Complete PT resolution	Cerebellar ischaemia resulting in ataxic gait
Santos-Franco et al. (30)	Stenting	Femoral access; systemic intravenous tirofiban.	DAPT post procedurally (unclear duration).	N/A	Complete symptom resolution at 24 h	1 month and 6 months	Complete PT resolution	None
Shastri et al. (31)	Stent-assisted coiling (*n* = 2)	Case 1: IJV access	DAPT for 2 weeks pre-procedure to 6 months. Thereafter, Aspirin monotherapy lifelong.	1 day	Immediate symptom resolution	4 weeks and 12 months	Complete PT resolution at both follow up reviews	None
		Case 2: Femoral access; No comment on heparinisation in either case.						
Signorelli et al. (32)	Balloon-assisted coiling	Femoral access; systemic intravenous heparinisation.	DAPT for 5 days prior to 3 months post procedure. Thereafter, aspirin monotherapy for 9 months.	N/A	Immediate symptom resolution	6 and 18 months	Complete PT resolution at both follow up reviews	None
Trivelato et al. (33)	Stent-assisted coiling	Left ICA access; systemic intravenous heparinisation.	DAPT for 5 days prior to procedure. No comment on duration post.	N/A	Immediate symptom resolution	16 months	Complete PT resolution	None
Walker et al. (4)	Stent-assisted coiling (*n* = 23)	Upper extremity venous or arterial access; no comment on heparinisation.	DAPT 1-week pre and 3 months post procedure.	1 day	Complete symptom resolution *n* = 16	3 months and 1 year	N/A	None
					Partial resolution *n* = 3			
					No resolution *n* = 4			
Zenteno et al. (34)	Stenting and Coiling	Femoral access; systemic intravenous heparinisation.	DAPT daily post-procedurally (unclear duration).	N/A	N/A	6 months	Complete PT resolution	None
Zur et al. (35)	Stent-assisted Woven Endobridge device deployment (intrasaccular device)	Femoral access; systemic intravenous heparinisation.	N/A	1 day	Immediate symptom resolution	N/A	N/A	None

This table summarises the endovascular treatment strategies reported across the included studies for the management of pulsatile tinnitus associated with venous sinus diverticula. Recorded domains include endovascular technique, technical parameters, peri-procedural management, length of hospital stay, treatment efficacy, complications, follow-up duration, and clinical outcomes. Across studies, symptom improvement—often complete—was frequently observed both immediately following the procedure and at follow-up. Procedural complications were uncommon, with most studies reporting short hospital stays and routine use of peri-procedural antiplatelet therapy to mitigate thromboembolic risk. Asterisks (*) denote items further clarified in the table footnotes.

Qiu et al. (28): Stenting of severe transverse sinus stenosis resulted in increased bone remodelling and reduction in diverticula size on follow up imaging.

Santos-Franco et al. (30): Stenting from the distal portion of transverse sinus to the sigmoid sinus excluded the diverticula and expanded stenosis of the transverse sinus.

Zenteno et al. (34): Two stage endovascular technique with initial stenting across diverticula neck followed by coil embolization as pulsatile tinnitus re-appeared intermittently 3 months after the index procedure.

DAPT, Dual antiplatelet therapy (aspirin and clopidogrel).

IJV, Internal jugular vein.

PT, Pulsatile tinnitus.

Endovascular access was reported by 19 studies (*n* = 50). Femoral venous access was used in 42% of cases (*n* = 21), while alternative approaches included upper extremity venous access in 46% (*n* = 23), direct internal jugular vein access in 10% (*n* = 5) and direct internal carotid arterial access in 2% of cases (*n* = 1). 17 studies (*n* = 47) outlined peri-procedural management including dual anti-platelet therapy in 87.23% (*n* = 41), aspirin only in 10.64% (*n* = 5) and anti-coagulant therapy in 2.13% (*n* = 1) of cases. Length of hospital stay was reported in eight studies, with a mean duration of 2.25 days. Clinical outcomes were reported in 22 studies (*n* = 77). Complete resolution of PT was observed in 84.4% of patients, partial improvement in 10.4%, and no improvement in 5.2%. Follow-up duration was reported in 19 studies and ranged from 4 weeks to 2 years. Procedural complications were described in 3 studies (*n* = 5) and included transient cerebellar ischaemia (*n* = 1), venous thrombosis (*n* = 1), arterial access haematoma, and coil stretching (*n* = 2).

## 4 Discussion

### 4.1 Summary of principal findings

To our knowledge, this scoping review represents the first comprehensive synthesis of the literature examining endovascular treatment of PT secondary to venous sinus diverticulum. This review collates and systematically maps existing evidence on diverticulum characteristics, endovascular treatment strategies, clinical outcomes, and reported complications across a heterogeneous body of literature predominantly comprising case reports, case series, and small retrospective observational studies.

Across the included studies, a range of endovascular techniques were employed, most commonly stent-assisted coil embolization, followed by coil embolization alone, venous sinus stenting, and, less frequently, intrasaccular flow disruption with the Woven EndoBridge device. Despite variability in anatomical presentation, procedural approach, and outcome assessment, endovascular intervention was consistently associated with high rates of symptomatic improvement and low reported complication rates. These findings are broadly consistent with prior reports suggesting that venous sinus diverticulum represents a treatable cause of PT and that endovascular approaches offer a minimally invasive therapeutic option (15). Building on this overall pattern of favourable outcomes, a more detailed appraisal of clinical effectiveness of endovascular options is warranted.

### 4.2 Clinical effectiveness and durability of endovascular treatment

All forms of endovascular treatment of venous diverticula were associated with high rates of clinical improvement, with resolution of PT constituting the primary measure of effectiveness. However, stent-assisted coil embolization was most widely utilised and reported on for its high success rates of symptom relief. In general, most patients experienced immediate or early symptom resolution following intervention, including those with prolonged or refractory symptoms, supporting a direct relationship between exclusion of the diverticulum and attenuation of pathological venous sound transmission (4, 15). Where follow-up data were available, symptomatic benefit was generally maintained, supporting the concept that durable symptom relief may arise from persistent modification of local venous biomechanics, including reduced flow turbulence, wall shear stress, and acoustic transmission following diverticular exclusion (27). Delayed or partial improvement occurred in a subset of specific cases, and may reflect progressive haemodynamic stabilisation or post-procedural vascular remodelling rather than technical failure (15, 22, 25, 30).

Clinical outcomes appeared to vary by diverticulum location. Higher rates of symptom resolution were reported for diverticula involving the transverse and sigmoid sinuses compared with those arising from the jugular bulb (4). This discrepancy may relate to anatomical complexity, local venous flow characteristics, or technical challenges in achieving effective lesion exclusion at the jugular bulb. Nonetheless, reports of successful treatment in this subgroup indicate that anatomical location alone does not preclude favourable outcomes, and that diverticular morphology and haemodynamic context are likely important contributors to treatment response (16, 33).

The contribution of upstream venous sinus stenosis, however, remains incompletely defined. Some literature suggests that venous sinus diverticulum is an acquired lesion as a consequence of stenotic vasculature (3). Hence, it is argued that targets of treatment should be the upstream stenosis itself, rather than the diverticular sac. While symptom resolution following sinus stenting alone has been described, some series have reported effective symptom control with diverticulum-directed treatment despite persistent upstream stenosis, supporting the diverticulum as a principal acoustic source (17). Given the absence of comparative data and the predominance of descriptive studies, these observations support an individualised approach to treatment selection informed by patient-specific anatomical and haemodynamic factors (4).

### 4.3 Anatomic variability and endovascular decision-making

Anatomical heterogeneity of venous sinus diverticula emerged as a central determinant of endovascular strategy across the reviewed literature. Variations in diverticular morphology, neck configuration, and parent sinus calibre influenced both device selection and technical approach. Saccular and lobulated diverticula were most amenable to coil embolization, whereas fusiform configurations posed greater technical challenges, particularly with respect to coil stability and the risk of parent sinus compromise (3, 17).

Differences in venous sinus diameter and geometric complexity, especially at the sigmoid sinus–jugular bulb junction, further constrained the applicability of standard coiling or stent-assisted techniques. In anatomically complex cases, alternative strategies including intrasaccular constructs—were employed to achieve diverticular exclusion without excessive device burden, although experience remains limited and largely case based (19).

Additional complexity arose in patients with multiple diverticula or highly irregular venous anatomy, necessitating tailored technical adaptations to enable effective treatment (16). Collectively, these findings highlight that endovascular management of venous sinus diverticula cannot be algorithmic. Rather, successful intervention depends on careful anatomical and haemodynamic characterisation, with procedural strategy adapted to individual morphology rather than dictated by a single preferred technique.

### 4.4 Safety profile and risk considerations

Endovascular treatment of venous sinus diverticulum was generally reported to have a favourable safety profile, with most patients experiencing no procedural complications. Across the reviewed literature, adverse events were uncommon, supporting endovascular intervention as a low-risk option for selected patients with PT secondary to venous diverticula. Nonetheless, the predominantly descriptive nature of the evidence and potential under-reporting of complications warrant cautious interpretation.

Risk profiles varied according to diverticular anatomy and treatment strategy. Wide-necked diverticula were associated with an increased risk of coil migration or herniation when treated with coil embolization alone (34), prompting the use of adjunctive stent placement in some cases (20). A comparison of the safety profile for upstream stenosis stenting, and direct stent-assisted coil embolization can be made. Regarding the former, prolonged antiplatelet therapy is often warranted post-procedure to mitigate the risk of stent-related venous thrombosis (28). However, stent-assisted coil embolization of the diverticular sac itself, has had isolated cases of ataxic gait secondary to cerebellar ischaemia (29).

Device selection may also influence procedural safety. While platinum coils were most commonly used, alternative coil technologies have been described that may facilitate denser packing and reduce procedural and fluoroscopy times, potentially improving safety, although comparative data are limited (16, 25, 31). Overall, these findings emphasise that procedural risk is not uniform and should be assessed in the context of individual anatomy, device choice, and patient-specific factors.

### 4.5 Positioning endovascular therapy Among treatment options

In light of its generally favourable safety profile and minimally invasive nature, endovascular therapy has assumed an increasingly important role in the management of PT associated with venous sinus diverticulum (36–38). However, it should be considered within a broader therapeutic landscape that includes conservative management and surgical intervention, with treatment selection informed by anatomical, clinical, and patient-specific factors (39–41).

Surgical approaches, most commonly transmastoid sigmoid sinus wall reconstruction or resurfacing, have demonstrated high rates of symptom resolution in published series, particularly in patients with focal diverticula or bony dehiscence amenable to direct repair (42–45). These techniques are associated with durable outcomes, but require open skull base exposure and carry recognised risks, including cerebrospinal fluid leak, cranial nerve injury, wound complications, and longer hospitalisation (46–49). Consequently, surgical management is often reserved for anatomically suitable cases or situations in which endovascular options are contraindicated or unsuccessful (50).

Endovascular treatment has therefore emerged as a less invasive alternative that addresses the presumed haemodynamic drivers of symptoms while avoiding open surgical exposure. Taken together, these considerations suggest that surgical and endovascular therapies should be viewed as complementary rather than competing modalities. Optimal management requires an individualised, multidisciplinary approach that integrates anatomical characteristics, haemodynamic assessment, procedural risk, and patient preference.

### 4.6 Strength and limitations

This scoping review was conducted using a rigorous and transparent methodological framework consistent with PRISMA-ScR guidance, enabling a comprehensive mapping of the literature on endovascular treatment of PT associated with venous sinus diverticulum. The use of a structured Population–Concept–Context approach, a multi-database search strategy, and independent screening and data extraction strengthens the completeness and reproducibility of the review. By systematically synthesising study-, patient-, and treatment-level data, this work provides a consolidated overview of endovascular techniques, clinical outcomes, and reported complications within an otherwise fragmented evidence base. The inclusion of a structured methodological quality appraisal further facilitates contextual interpretation of the findings.

However, several limitations merit consideration. Firstly, the available evidence is derived predominantly from case reports and small case series, with limited cohort-level data, restricting comparative analysis and precluding formal assessment of treatment effectiveness. Secondly, outcome definitions and assessment methods were heterogeneous, follow-up durations were variable, and tinnitus-specific validated outcome measures were infrequently used, limiting evaluation of durability and patient-centred benefit. Thirdly, selection and publication bias are likely, given the rarity of the condition and the predominance of favourable outcome reporting. Finally, restriction to peer-reviewed, English-language publications may have resulted in the exclusion of relevant studies.

### 4.7 Implications for clinical practice and future research

The findings of this scoping review suggest that endovascular treatment is a viable management option for selected patients with PT attributable to a venous sinus diverticulum, particularly when venous anatomical abnormalities are well characterised and alternative causes have been excluded. In clinical practice, these data support consideration of endovascular approaches within a multidisciplinary framework involving neurology, otology, and neurointerventional expertise, with careful patient selection, informed consent, and counselling regarding the limited quality of existing evidence. Of note, standardised diagnostic pathways incorporating high-resolution venous imaging and haemodynamic assessment may help refine treatment selection and improve outcome consistency.

From a research perspective, the predominance of descriptive studies highlights the need for higher-quality evidence. Future investigations should prioritise prospective, multicentre designs with standardised reporting of venous anatomy, procedural technique, and peri-procedural management. The systematic use of validated tinnitus-specific outcome measures and longitudinal follow-up is also essential to assess symptom durability and patient-reported benefit. Comparative studies evaluating endovascular vs. surgical or conservative strategies, as well as investigations exploring predictors of treatment response and complication risk, will be critical to inform evidence-based practice and guide the development of clinical consensus in this evolving field.

## 5 Conclusions

Our study demonstrates that endovascular treatment of PT associated with venous sinus diverticulum is associated with high rates of symptomatic improvement and low reported complication rates in carefully selected patients. However, the available evidence is derived predominantly from case reports and small case series, with limited comparative data, heterogeneous outcome assessment, and variable follow-up durations. Robust prospective, multicentre studies incorporating standardised diagnostic criteria, validated tinnitus-specific outcome measures, and longer-term follow-up are required to define optimal patient selection, comparative effectiveness, and durability of endovascular interventions.
